# COVID-19 outbreaks among isolated Amazonian indigenous people, Ecuador

**DOI:** 10.2471/BLT.20.283028

**Published:** 2021-07-01

**Authors:** Aquiles R Henriquez-Trujillo, Esteban Ortiz-Prado, Ismar A Rivera-Olivero, Nemonte Nenquimo, Andrés Tapia, Mitchell Anderson, Tannya Lozada, Miguel Angel Garcia-Bereguiain

**Affiliations:** a One Health Research Group, Universidad de Las Américas, Via a Nayón S/N.170125, Quito, Ecuador.; b Organización Waorani de Pastaza, Shell, Ecuador.; c Nacionalidad Kichwa de Pastaza and Confederation of Indigenous Nationalities of the Ecuadorian Amazon, Puyo, Ecuador.; d Amazon Frontlines, San Francisco, United States of America.

By October 2020, Ecuador had more than 166 excess deaths per 100 000 population, becoming one of the countries with the highest number of coronavirus disease 2019 (COVID-19) related deaths.^1^^–^^3^ Official data at the time of the study reported more than 170 000 confirmed COVID-19 cases and more than 8000 related deaths; however, these numbers are expected to be much higher.^3^ Severe acute respiratory syndrome coronavirus 2 (SARS-CoV-2) diagnosis has mainly targeted large urban areas such as Guayaquil, Quito or Cuenca, the only cities where free testing is available through the National Institute of Public Health Research. Despite the institute’s efforts to improve testing capacity,^4^ no more than 3000 polymerase chain reaction (PCR) tests have been performed per day in Ecuador, for a population of 17 million.^5^

Here we describe the impact of COVID-19 on the neglected indigenous communities of the Ecuadorian Amazonia. The low testing capacity, urban location of laboratories and high prices (90–120 United States dollars) of PCR tests makes SARS-CoV-2 testing hard to access for rural and underserved communities, including traditionally neglected indigenous people, who represent an estimated 7% of the Ecuadorian population. Amazonian indigenous communities are highly vulnerable to public health threats such as COVID-19 because of their extreme isolation and the poor health infrastructure in the areas they inhabit.^3^

The Confederation of Indigenous Nationalities of the Ecuadorian Amazon reported outbreaks of COVID-19 from June to July 2020 in these communities. This nongovernmental organization contacted our research group at Universidad de Las Americas, a private academic institution, to deploy teams to provide medical assistance and SARS-CoV-2 testing by PCR.^6^^–^^11^ The Waorani, Siona, Kichwa, Shuar and Kofan people, some of the most isolated groups in the area and often only accessible by boat or aeroplane, were included in this intervention from June to September 2020.

We found COVID-19 outbreaks in 12 out of 14 the communities visited, with only two remaining SARS-CoV-2 free. A total of 769 individuals were tested (that is, most members from the communities included in the intervention) enrolled in the sampling. SARS-CoV-2 attack rates ranged from 30% to 90% in those communities, and the overall percentage of infected people was 49% (378/769). SARS-CoV-2 positivity, per ethnic group, was 46.0% (70/152) for Waoranis; 31.5% (40/127) for Sionas; 49.8% (124/249) for Kichwas; 54.9% (45/82) for Kofans; and 62.3% (99/159) for Shuars. We did not record any deaths during our visits. While community leaders said that some deaths were potentially related to COVID-19, the number of COVID-19 deaths is uncertain.^3^ COVID-19 outbreaks in the Amazonian indigenous communities are particularly worrying for the Tagaeri and Taromenane people, the last uncontacted groups of the Ecuadorian Amazonia at the Untouchable Zone of Yasuní National Park, where access is restricted due to the presence of uncontacted Amazonian tribes. These communities have some contact with the Waorani people. 

Our coordinated actions with Waorani leaders to report COVID-19 outbreaks in their communities supported the lawsuit against Ecuador’s President and Vice President, for having failed to provide these communities with protection against COVID-19.^12^ In a public statement during the lawsuit, Waorani leaders said that their actions were aimed at protecting their elders or *Pikenani*, as well as their uncontacted relatives.^12^ The Provincial Court of Pichincha recently ruled in favour of the Waorani people and required the Government of Ecuador to conduct SARS-CoV-2 tests and take action to contain the virus in Waorani territory.^12^

Today, SARS-CoV-2 testing capacities have not yet improved in Ecuador and COVID-19 vaccination programmes have been progressing slowly. Moreover, the effect of COVID-19 on the indigenous communities described in this study has been reported for other indigenous communities in Ecuador as well; they also have excess death rates due to COVID-19.^3^ We call on the Government of Ecuador to act to improve the court-mandated measures to protect the Waorani people, as well as all the Amazonian, Andean and Coastal ethnic groups. We also call on all governments in the continent to protect the indigenous people of the Americas against COVID-19.
